# Moco Carrier and Binding Proteins

**DOI:** 10.3390/molecules27196571

**Published:** 2022-10-04

**Authors:** Tobias Kruse

**Affiliations:** Institute of Plant Biology, Technische Universität Braunschweig, 38106 Braunschweig, Germany; t.kruse@tu-bs.de

**Keywords:** molybdenum cofactor, molybdenum cofactor carrier protein, molybdenum cofactor binding protein, Moco

## Abstract

The molybdenum cofactor (Moco) is the active site prosthetic group found in numerous vitally important enzymes (Mo-enzymes), which predominantly catalyze 2 electron transfer reactions. Moco is synthesized by an evolutionary old and highly conserved multi-step pathway, whereby the metal insertion reaction is the ultimate reaction step here. Moco and its intermediates are highly sensitive towards oxidative damage and considering this, they are believed to be permanently protein bound during synthesis and also after Moco maturation. In plants, a cellular Moco transfer and storage system was identified, which comprises proteins that are capable of Moco binding and release but do not possess a Moco-dependent enzymatic activity. The first protein described that exhibited these properties was the Moco carrier protein (MCP) from the green alga *Chlamydomonas reinhardtii*. However, MCPs and similar proteins have meanwhile been described in various plant species. This review will summarize the current knowledge of the cellular Moco distribution system.

## 1. Introduction

**Figure 1:**
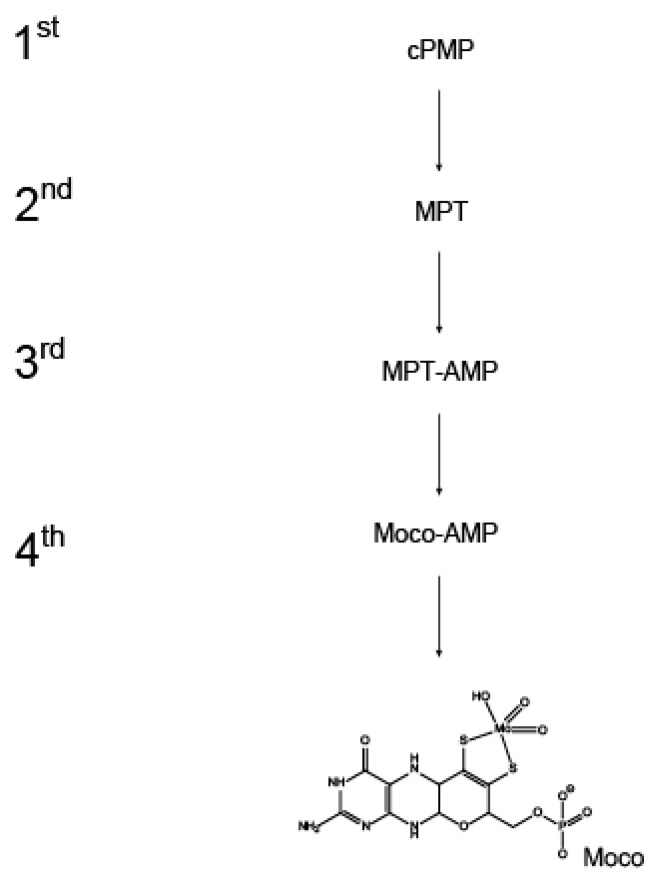
The eukaryotic molybdenum cofactor biosynthesis. Molybdenum cofactor (Moco) biosynthesis comprises four subsequent reaction steps, each giving yield to the intermediates shown: The reaction product of the first reaction step is cyclic pyranopterin monophosphate (cPMP) which is subsequently converted to molybdopterin (MPT) in the second reaction step. MPT is adenylyated, yielding MPT-AMP that is then converted to Moco-AMP. In the ultimate, fourth reaction step, the phosphoanhydride-bond within Moco-AMP is hydrolyzed and Moco is released.

The molybdenum cofactor (Moco) biosynthesis pathway comprises several strictly conserved reaction steps, ultimately yielding Moco. Within the first step of the pathway, GTP is converted to cyclic pyranopterin monophosphate (cPMP, [1,2]) a reaction thatinvolves the formation of the instable intermediate 3′,8-cH_2_GTP [3,4]. Recent work suggests that it is the first step of eukaryotic Moco biosynthesis which is under regulatory control as deduced from the finding that in the filamentous fungus *Neurospora crassa* (*N. crassa*, [5]) the 3′,8-cH_2_GTP synthesizing enzyme is co-regulated (precisely the formation of one particular splice variant) with the major *N. crassa* Moco user enzyme (i.e. nitrate reductase). In the second step of the pathway, cPMP is converted to molybdopterin (MPT) in a stoichiometric reaction which is catalyzed by the heterotetrameric MPT synthase complex. This reaction is best characterized by the fact that the MPT dithiolene sulfurs are incorporated here to assemble the metal-free Moco scaffold [6,7,8]. In the subsequent third and fourth reaction steps, molybdate is incorporated into the MPT dithiolene moiety (for a recent review, see [9] in this Special Issue). Initially, MPT binds to the molybdenum insertase (Mo-insertase) G-domain where it becomes adenylylated, yielding MPT-AMP (termed adenylated MPT, [10]). In the following, MPT-AMP is used as the substrate from the Mo-insertase E-domain, which catalyzes the insertion of molybdate, yielding Moco-AMP [11]. The subsequent hydrolysis of the Moco-AMP phophoanhydride-bond then releases Moco (Figure 1, [11]), which can now be transferred either to the Moco-dependent enzymes or to the cell’s Moco transfer and storage system [12].

For a recent review of Moco biosynthesis, see Ralf Mendel’s review in this Special Issue [13]. The most likely candidate proteins for the cellular transfer and storage system have been identified and characterized in plants (i.e., Moco carrier protein (MCP, [14,15,16,17]) and Moco binding proteins (MoBP, [18,19]). However, these proteins possess considerably different Moco binding and stabilizing properties [12,18,19], as documented by the finding that plant MCP proteins can be co-purified with significant amounts of Moco [16,17], while MoBP proteins can’t [18,19]. Notably, the hitherto sole known prokaryotic MCP was found to be co-purified with comparably low amounts of Moco [20], thus classifying the Moco binding properties of this protein to be similar to the *A. thaliana* MoBP proteins.

**Figure 2:**
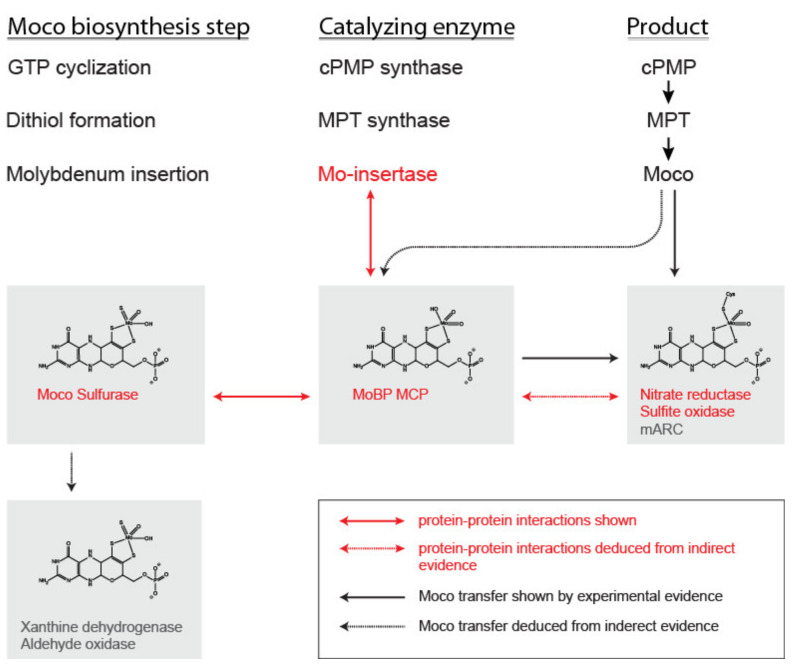
Molybdenum cofactor synthesis and transfer. The molybdenum cofactor (Moco) biosynthesis reaction steps, catalyzing enzymes, and products are given in the upper third of the figure. The molybdenum insertase (Mo-insertase) is the platform enzyme of the biosynthesis complex and interacts with the cellular transfer system (i.e., Moco binding proteins (MoBP) and putatively also with Moco carrier proteins (MCP)) as well as with nitrate reductase and sulfite oxidase [21]. The interactions of the Mo-insertase with MoBP proteins and the interaction of these proteins with the Moco sulfurase and nitrate reductase [18] are indicated by red arrows. The interaction of MoBP proteins with nitrate reductase and the plant Mo-insertase Cnx1 has been studied in [18]. Dashed lines indicate indirect evidence for the interaction, while solid lines indicate direct evidence. The transfer of Moco from source to sink is indicated by black arrows. Again dashed lines indicate indirect evidence, while solid lines indicate direct evidence for Moco transfer.The interaction network of *Arabidopsis thaliana* (*A. thaliana*) MoBP proteins within the cell has been studied in [21], while the interaction of MCP with the Mo-insertase has not been studied as of yet. Upon synthesis, Moco can be transferred on plant sulfite oxidase in vitro [23]. Indirect evidence for the interaction of *C. reinhardtii* MCP with Moco free nitrate reductase, present in the *nit-1* system was described in [14]. Moco binding proteins were shown to enhance the formation of functional nitrate reductase assembly in the *nit-1* system [18], while *C. reinhardtii* as well as *V. carteri* MCP proteins were found to serve as Moco donors here [16,17]. No evidence for the transfer of Moco from MoBP proteins to the (plant) Moco sulfurase ABA3 has been described in the literature as yet. The transfer of sulfurated Moco from ABA3 to the enzymes of the Xanthine oxidase family can be assumed.

Next to the biochemical characterization, the results coming from in vivo approaches also classify the MoBP proteins to function within the cellular Moco transfer and storage system. Members of the MoBP protein family were shown to interact with both the molybdenum insertase [21], the Mo-enzyme nitrate reductase [18,21], and the Moco sulfurase ABA3 in plants [21]. However, for MCP proteins, a similar detailed in vivo characterization is missing, but these were shown to reconstitute nitrate reductase activity both in fully defined in vitro systems and in the *nit-1*-based reconstitution assay [14,16,22]. Within this review, the current view on the function of MCP and MoBP proteins for the cellular Moco transfer will be summarized (see Figure 2 for an overview).

## 2. Identification and Occurance of Moco Carrier and Binding Proteins

The first incidence ofthe existence of a Moco carrier protein (MCP) was reported for the prokaryote *E. coli* in 1979 [24]. However, indications for the existence of an MCP protein were also later on reported for eukaryotes [14,25], while—similar to *E. coli*—he identity of the protein was not elucidated in the initial study. However, for *C. reinhardtii* [14] following work revealed the identity of the MCP amino acid sequence and the encoding gene [15]. Work with the recombinant *C. reinhardtii* MCP protein then characterized its Moco-binding properties [15,16] and revealed its structure [16]. Having hands on the structure was of great importance since, at that time, the identification of *C. reinhardtii* MCP homologs was restrained to the use of structure-based homology searches, as no meaningful results were obtained by using the *C. reinhardtii* MCP amino acid sequence as a query in pBLAST searches [18]. Structure-based homology searches then revealed two homologous eukaryotic (plant, *Arabidopsis thaliana*) structures [18] and three of bacterial origin [16]. However, the identified *A. thaliana* proteins were found to be part of a protein family [26] and were termed Moco binding proteins (MoBP) to indicate their — as compared to *C. reinhardtii* MCP—different Moco-binding properties. Notably, next to the existence of an MCP in *C. reinhardtii* also a putative MoBP was identified here, which is not member of a protein family [19]. The following work took advantage ofthe ongoing progress in the field of genome sequencing and annotation (Table 1, [17,20])**,** which allowed it to identify potential *C. reinhardtii* MCP homologs amino acid sequence-based in the algae *Chlamydomonas eustigma*, *Haematococcus lacustris*, *Gonium pectoral* and *Volvox carteri* [17]. Likewise, *C. reinhardtii* MCP homologs were identified in various bacteria [20], with candidate proteins from the phylum *Cyanobacteria* and α*-Proteobacteria* showing the highest degree of sequence identity to CrMCP (ranging from 50–60%, [20]).

## 3. Biochemical and Structural Characterization of Moco Carrier and Binding Proteins

Upon identification of the *C. reinhardtii* MCP encoding gene (*mcp1*) it became possible to express and purify the recombinant protein, facilitating the biochemical characterization of MCP [15,16]. Doing so revealed the recombinant protein to be a tetramer in solution, and further, *C. reinhardtii* MCP was found to be co-purified with Moco upon expression in the *E. coli* TP1000 strain [27], while no MPT was co-purified with recombinant *C. reinhardtii* MCP upon expression in the MPT accumulating *E. coli* RK5206 strain [28]. Most importantly, next to the capability to bind Moco, the recombinant *C. reinhardtii* MCP was found to serve as a Moco donor for Moco-free nitrate reductase in *nit-1* cell extracts without preceding heat or acidic treatment [16]. As this renders *C. reinhardtii* MCP to bind Moco reversible, it was concluded that within the cell the carrier protein could serve as a Moco storage protein to buffer a varying Moco demand [15,16]. To give a first insight into the molecular detail behind *C. reinhardtii* MCP Moco-binding, a putative Moco binding site was identified and a docking approach suggested the positioning of Moco within here. Adjacent to the putative binding site, a structural disorder loop is located. Upon the sequence-based identification of various other putative eukaryotic MCPs, within the N-terminal part of the MCP amino acid sequence, a sequence stretch of low homology (lhr) was identified [17]. For *C. reinhardii* MCP, this sequence was found to form the aforementioned structurally disordered loop and it was suggested that the observed differences in sequence result in varying properties of the binding site, which were suggested to be required for the adaption of each particular MCP to the varying cellular properties of the respective host [17]. Consistently, the (eukaryotic) MCP from *Volvox carteri* (*V. carteri*) was found to adapt the Moco binding properties from the prokaryotic MCP from *Rippkaea orientalis*, when the *V. carteri* lhr sequence is replaced with the *R. orientalis* lhr sequence [17]. Next to recombinant MCPs, also the MoBP proteins from *A. thaliana* were characterized biochemically, and contrary to the recombinant *C. reinhardtii* and *V. carteri* MCPs [16,17], their characterization revealed none of them to be co-purified with significant amounts of Moco, albeit in vitro loading of Moco to MoBP proteins was possible [18]. The Moco K_D_ values determined for the recombinant MoBP proteins were found to lay in the low µM range thus being in the range of the Moco K*_D_* value determined for recombinantCnx1E [29]. As both, recombinant Cnx1E and recombinant MoBP proteins cannot be co-purified with significant amounts of Moco, it can be concluded that the Moco K*_D_* values determined do not allow the co-purification of Moco with any of these proteins. Next to the rather low Moco K*_D_* values recorded [18], the fact that member(s) of the MoBP protein family were found to interact with the Mo-insertase Cnx1 (catalyzing the final step of Moco biosynthesis [18,30] and downstream Moco user enzymes (i.e., nitrate reductase [18] and Moco sulfurase [30]) led to the conclusion that these proteins have a function for Moco transfer in the cell but not for Moco storage (see also [19]). Likewise, for *C. reinhardtii* MCP data were reported that also suggests a protein–protein interaction here with a Moco user enzyme (i.e., nitrate reductase, [14]) which was shown to be of functional importance. Consequently, MCP proteins were suggested to be involved in both, Moco transfer and storage [19]. Clearly, one of the major differences between MoBP and MCP proteins is the varying affinity for Moco binding, which could be seen as an adaption to fulfill the respective cellular function(s) these proteins are assumed to have.

The first structurally characterized Moco carrier protein was the MCP from *C. reinhardtii* [16]. The *C. reinhardtii* MCP monomers are arranged in a Rossmann-like fold and the symmetric homo-tetramer assembled from these was described as dimers of dimers, which was traced back to the fact that each monomer contributes to two distinct intermolecular interactions here [16]. Each monomer houses one putative Moco binding site. Recently the structure of the first prokaryotic (*Rippkaea orientalis*, *R. orientalis*) MCP has been reported [20], which was found to be highly similar to that of *C. reinhardtii* MCP. Similar to *C. reinhardtii* MCP and also for *R. orientalis* MCP, a positively charged surface depression was identified as a putative Moco binding site and consistent with the docking results reported for *C. reinhardtii* MCP [16], likewise for *R. orientalis* MCP, the phosphate moiety of Moco was suggested to reside deeply buried within the binding pocket while the molybdenum center was suggested to be rather surface exposed. The *C. reinhardtii* monomer was found to be structurally highly similar to that of the MoBP monomer, while other than MCP proteins, MoBP proteins form dimers both in the structure and in solution [18] and contrary to MCP, Moco binding to *Arabidopsis* MoBP proteins was traced back to the C-terminus of the protein [18], which potentially could explain the differences in Moco binding displayed by MoBP and plant MCP proteins. 

## 4. Conclusions

The fate of Moco upon synthesis can only be traced back to limited detail. This is due to the finding that—contrary to Moco biosynthesis—no mutants of the cellular Moco distribution and storage system are known. However, candidate proteins were identified in plants and bacteria, while hitherto, in the animal and fungal kingdoms homologs to the plant proteins have not been revealed and characterized so far. Therefore it can be concluded that though Moco biosynthesis is strictly conserved amongst the kingdoms of life, it´s trafficking and most likely also the processes underlying the insertion of Moco into the cognate molybdenum enzymes is not. It is tempting to assume that the presence of MCP/MoBP proteins in plants is linked to the outstanding importance of nitrate reductase here. However, fungi also possess the nitrate assimilation pathway but lack functional homologs to the known Moco carrier and binding proteins, suggesting that here the Moco carrier/binding proteins were lost during evolution. This could be explained by the fact that Moco biosynthesis is tightly regulated here, thus rendering a Moco storage system needless.

## Figures and Tables

**Table 1:** Moco carrier and Moco binding proteins. The accession numbers of known and characterized Moco carrier (MCP) and Moco binding proteins (MoBP) and the corresponding publication where these were first described are tabulated. Ataya 2003 [15], Kruse 2010 [18], Krausze 2020 [20], Hercher 2020 [17].

Accession Number	Annotation	Publication	Organism
AY039706	CeMcp1	[15]	*Chlamydomonas reinhardtii*
At2g28305	MoBP1	[18]	*Arabidopsis thaliana*
At5g11950	MoBP2		
At2g37210	MoBP3		
At4g35190	MoBP4		
At3g53450	MoBP5		
At5g03270	MoBP6		
WP_012595913	RoMCP	[20]	*Rippkaea orientalis*
XP_002954772.1	VcMCP	[17]	*Volvox carteri*

## Data Availability

Not applicable.
